# Perceptual estimation obeys Occam's razor

**DOI:** 10.3389/fpsyg.2013.00623

**Published:** 2013-09-23

**Authors:** Samuel J. Gershman, Yael Niv

**Affiliations:** ^1^Department of Brain and Cognitive Sciences, Massachusetts Institute of TechnologyCambridge, MA, USA; ^2^Department of Psychology and Princeton Neuroscience Institute, Princeton UniversityPrinceton, NJ, USA

**Keywords:** perception, Bayesian inference, simplicity, unsupervised learning, categorization

## Abstract

Theoretical models of unsupervised category learning postulate that humans “invent” categories to accommodate new patterns, but tend to group stimuli into a small number of categories. This “Occam's razor” principle is motivated by normative rules of statistical inference. If categories influence perception, then one should find effects of category invention on simple perceptual estimation. In a series of experiments, we tested this prediction by asking participants to estimate the number of colored circles on a computer screen, with the number of circles drawn from a color-specific distribution. When the distributions associated with each color overlapped substantially, participants' estimates were biased toward values intermediate between the two means, indicating that subjects ignored the color of the circles and grouped different-colored stimuli into one perceptual category. These data suggest that humans favor simpler explanations of sensory inputs. In contrast, when the distributions associated with each color overlapped minimally, the bias was reduced (i.e., the estimates for each color were closer to the true means), indicating that sensory evidence for more complex explanations can override the simplicity bias. We present a rational analysis of our task, showing how these qualitative patterns can arise from Bayesian computations.

## Introduction

The fourteenth century English friar and theologian William of Occam advised philosophers “not to multiply entities beyond necessity” (Boehner, 1957). The contemporary interpretation of Occam's razor is that, all other things being equal, simpler explanations of data should be preferred to more complex explanations. This heuristic notion has found mathematical expression in Bayesian statistics (Jaynes, 2003) and algorithmic information theory (Li and Vitányi, 2008). It has since been applied to cognitive psychology as the “simplicity principle” (Chater and Vitányi, 2003; Feldman, 2003): the idea that humans seek simple explanations of their sensory input. Our focus in this paper is on unsupervised category learning, where evidence suggests that humans assign stimuli to a small set of categories, only inventing new categories when the stimulus statistics change radically (Anderson, 1991; Clapper and Bower, 1994; Pothos and Chater, 2002; Love et al., 2004; Sanborn et al., 2010).

If the categories people invent dictate how they “carve nature at its joints” (i.e., divide the environment into meaningful entities; see Gershman and Niv, 2010, then effects of Occam's razor should be discernible in perceptual estimation. Substantial evidence exists that categories shape perception (Huttenlocher et al., 1991, 2000; Goldstone, 1995; Hemmer and Steyvers, 2009). For example, Goldstone (1995) had participants judge the color of numbers and letters that varied in color along a red-violet gradient, and showed that stimuli belonging to the letter category (with typically red objects) were judged to be more red than identically colored stimuli belonging to the other category. As another example, syllables belonging to different phonetic categories are more easily discriminated than syllables with the same physical difference but belonging to the same category—the so-called *perceptual magnet effect* (Liberman et al., 1957). However, these studies assume a pre-defined category structure, whereas many real-world learning situations (particularly during development) require one to discover the underlying category structure from undifferentiated sensory data. In these situations, we expect that Occam's razor will influence the number of perceptual categories inferred from sensory data, and in turn govern participants' estimates of stimulus properties. The experiments reported in this paper were designed to test this hypothesis.

**Figure 1:**
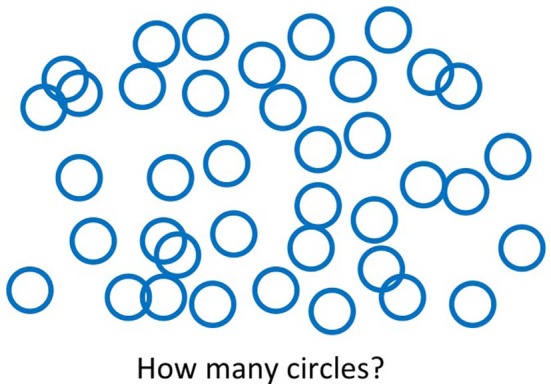
**Example trial**. On each trial, participants were presented with a random scattering of circles and asked to estimate the number of circles. The circles on each trial were all of the same color, and the number of circles was drawn from a color-specific Gaussian distribution.

The stimuli in our experiments consisted of randomly scattered colored circles displayed on a computer screen (Figure 1), similar to stimuli used in studies of number perception (Izard and Dehaene, 2008). Each trial was characterized by one of two colors, and all circles were displayed in this color. The number of circles on each trial was drawn from a color-specific Gaussian distribution. The distributions differed in their means (Experiments 1 and 3) or variances (Experiment 2). Participants were asked to judge how many circles there were on the screen, but did not have enough time to count them explicitly.

If the distributions corresponding to the two colors overlap sufficiently, Occam's razor dictates that the stimuli should all be assigned to one category despite their obviously different colors, a prediction formalized in several models of categorization (Anderson, 1991; Sanborn et al., 2010). The consequence of merging the two perceptual categories is that estimates will be “regularized” toward the average of the two distributions. In contrast, reducing overlap between the distributions is expected to diminish this regularization, as it supports separate categories for each color. Each of the experiments reported below included a high overlap condition in which merging (and hence more regularization) was expected to occur, and a low overlap condition in which splitting (and less regularization) was expected to occur.

To make our theoretical account explicit and quantitative, we present a computational model of human performance in our task. In the spirit of the probabilistic motivation for Occam's razor described above, we derive our model from hypothesized probabilistic assumptions about the environment and suggest that participants perform approximately optimal inference. In other words, we undertake a “rational analysis” (Anderson, 1990). Our aim is to elucidate the computational constraints, rather than particular processing or implementational mechanisms, that govern perceptual estimation in our task. We compare the rational model to an exemplar model (Medin and Schaffer, 1978; Nosofsky, 1986, 1988; Kruschke, 1992) which represents each data point as a unique perceptual category, and thus lacks a simplicity bias. Through quantitative model comparison, we show that the rational model is able to better account for our data.

## Experiment 1

**Figure 2:**
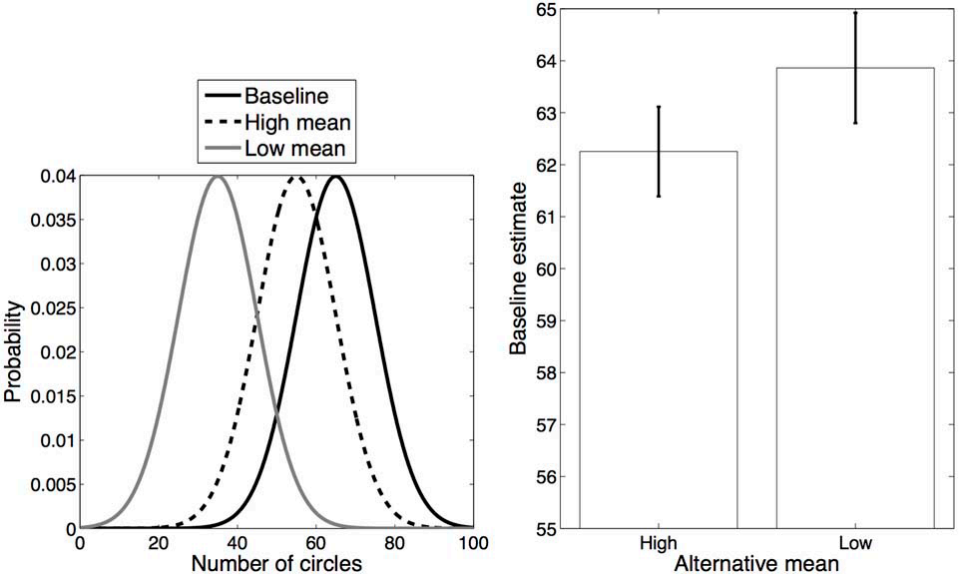
**Experiment 1 design and results. Left**: Distributions for each category. **Right**: Average estimates for the baseline category in each condition. Error bars represent standard error of the mean.

In our first experiment, we manipulated categorical overlap by varying (within subject) the distance between the means of the two distributions in blocks. Each block included one distribution (mean 65, standard deviation 10) which was designated the “baseline,” and a second, “alternative” distribution that either had low overlap (mean 35, standard deviation 10) or high overlap (mean 55, standard deviation 10) with the baseline distribution (Figure 2, left). We refer to these conditions as *Low mean alternative* and *High mean alternative*, respectively. In each block each of the distributions (alternative and baseline) was associated with a unique color, and circles appeared in that color on those trials in which the number of circles was drawn from that distribution.

Our instructions to participants made no mention of color. However, we expected participants to use color as a cue for categorization. More precisely, we expected use of the color cue to depend on a combination of sensory evidence (i.e., the number of circles) and a simplicity bias toward fewer categories. On High alternative mean blocks in which all trials had relatively similar numbers of circles, we expected participants to treat all trials as if they were one category, and effectively ignore color as a categorization cue. As a result, in these blocks we expected estimates about the number of circles to be affected by the statistics of both colors. In contrast, in Low alternative mean blocks in which there was less overlap between the number of circles in trials of one color as compared to the other color, we expected participants to treat each color as a separate category. If participants indeed learned separate estimates for each color, their estimates would be closer to the true mean of each of the distributions. As such, across blocks we predicted that estimates on the baseline trials would be lower on average in the High mean alternative condition than in the Low mean alternative condition, due to the regularization induced by merging the color categories together in the High mean but not in the Low mean alternative condition. Note that if participants ignored color and grouped all trials together on all blocks, we would expect the opposite: baseline estimates in the High mean alternative condition should be systematically higher than in the Low mean alternative condition. Alternatively, if participants always used color as a categorization cue, there should be no difference between estimates of baseline trials in the two conditions, since the baseline distribution is the same in both cases.

### Materials and methods

#### Participants

Fourteen students participated in the experiment for course credit or monetary compensation ($10). All subjects gave informed consent and the study was approved by the Princeton University Institutional Review Board.

#### Procedure

Stimuli consisted of colored circles displayed in a random spatial configuration within a bounded section of the computer screen. On each trial, the participant was presented with a pattern of randomly scattered (occasionally overlapping) circles (Figure 1), where the number of circles was drawn from a Gaussian with a category-specific mean and variance. There were two trial types: “baseline” trials in which the number of circles was drawn from a Gaussian with mean 65 and standard deviation 10), and “alternative” trials. In the “High mean alternative” block the latter trials were drawn from a Gaussian with mean 55 and standard deviation 10. In the “Low mean alternative” block, the alternative trials were drawn from a Gaussian with mean 35 and standard deviation 10. In all cases, the number of circles was truncated between 10 and 100, and rounded to the nearest integer. The two categories in each block were associated with a different color of circles (randomly chosen).

The participant was given 5 s to enter a two-digit estimate of the number of circles on the screen using the keyboard; if no response was entered within this time limit, a message indicated that the response was too slow and the trial was subsequently not used in data analysis. The circles remained on the screen during the 5 s response interval. After entering a response, the participant received feedback indicating the correct number of circles. Each subject performed eight blocks of the High mean alternative condition and eight blocks of the Low mean alternative condition (randomly interleaved), with 20 trials in each block (10 baseline and 10 alternative, randomly interleaved). All experiments were implemented in Matlab (Version 7.9.0.529) using the Psychophysics toolbox (Brainard, 1997).

We used paired-sample, two-sided *t*-tests to compare conditions. Effect sizes were measured using Cohen's *d*. We excluded subjects whose average errors (in terms of distance from the true mean) on alternative trials were greater than two standard deviations from the mean across all three experiments. No subjects were excluded from Experiment 1.

### Results and discussion

The average responses on baseline trials in each condition are shown in Figure 2 (right). Estimates of the number of circles on baseline trials in the High mean alternative condition (mean = 62.25) were significantly lower than in the Low mean alternative condition (mean = 63.86) [*t*_(13)_ = 2.41, *p* < 0.05, *d* = 0.64]. Moreover, the estimates were significantly lower than the true average in the High mean condition [*t*_(13)_ = 3.19, *p* < 0.05, *d* = 0.85] but not in the Low mean condition (*p* = 0.30). These results are consistent with the hypothesis that participants are more likely to assign the alternative and baseline distributions to the same category in the High mean alternative condition than in the Low mean alternative condition, due to greater overlap between the distributions in the former but not in the latter.

We also examined the estimates on alternative trials. The average number of circles reported by participants closely tracked the true average: 55.44 for the High mean alternative condition and 36.47 for the Low mean alternative condition. *T*-tests confirmed that average participant estimates were not significantly different from the true average (*p* = 0.51 for the High mean alternative condition and *p* = 0.07 for the Low mean alternative condition).

**Figure 3:**
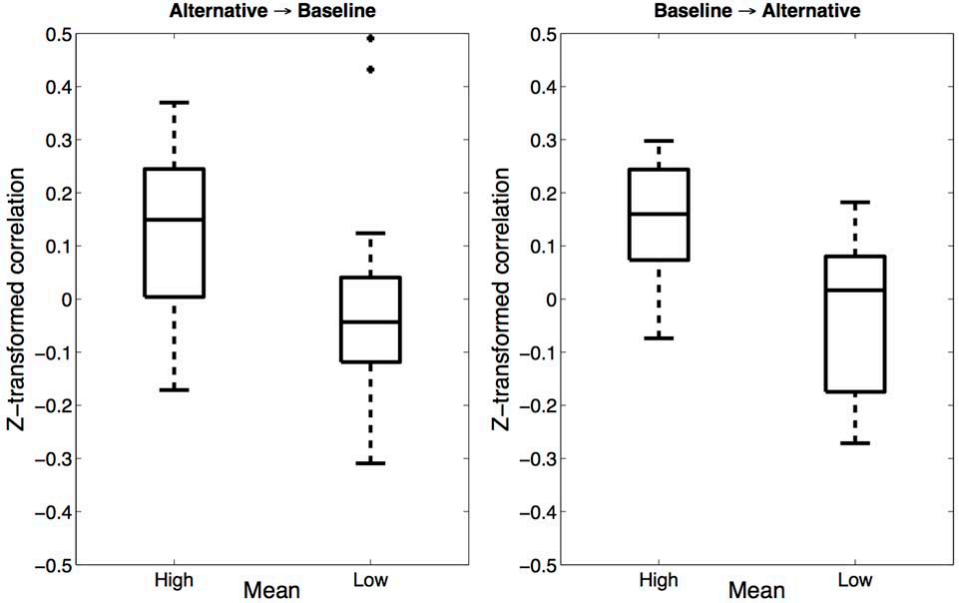
**Trial-wise correlations in Experiment 1**. **Left**: Fisher z-transformed correlations between estimates on baseline trials and on the preceding alternative trials. **Right**: Correlations between alternative trials and the preceding baseline trials.

If participants indeed merged the baseline and alternative categories in the High mean alternative condition, one might argue that we should also have seen regularization effects on the alternative trials. While we saw no evidence for such regularization in the trial-averaged data, it may be the case that regularization effects operate over timescales that are shorter than a whole block. To test this hypothesis, we calculated the correlation between estimates on each baseline trial and the preceding alternative trial (note that, due to the randomized trial order, the preceding alternative trial might have been several trials back). We reasoned that if estimates are influenced by recently experienced trials, then the correlation dependent measure should be positive. Importantly, this should only occur if both trials were assigned to the same merged category. Figure 3 (left) shows the results of this analysis: Fisher z-transformed correlations were significantly greater than 0 in the High mean alternative condition [*t*_(13)_ = 3.14, *p* < 0.01, *d* = 0.84] but not in the Low mean alternative condition (*p* = 0.86). We also examined the influence of baseline trials on subsequent alternative trials (Figure 3, right): Again, Fisher z-transformed correlations were significantly greater than 0 in the High mean alternative condition [*t*_(13)_ = 4.47, *p* < 0.001, *d* = 1.19] but not in the Low mean alternative condition (*p* = 0.50). These results are consistent with the hypothesis that the High mean alternative condition promotes category merging while the Low mean alternative condition does not.

The correlation analyses reported above also rule out an alternative explanation of our findings in terms of contrast effects. According to this explanation (see Holland and Lockhead, 1968), contrast between the baseline and alternative categories is accentuated in the Low mean alternative condition, causing participants to produce higher estimates for baseline trials compared to estimates in the High mean alternative condition. Such a contrast explanation would predict *negative* correlations between estimates in the baseline and alternative trial types; yet we found no evidence for negative correlations.

## Experiment 2

**Figure 4:**
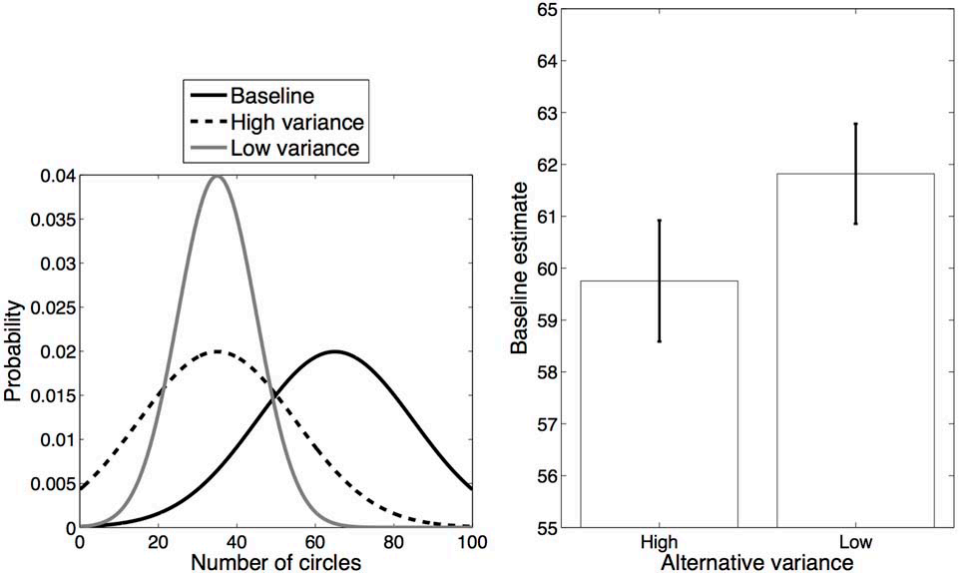
**Experiment 2 design and results**. **Left**: Distributions for each category. **Right**: Average estimates for the baseline category in each condition. Error bars represent standard error of the mean.

Our second experiment was identical to Experiment 1 in all respects except that we manipulated the variances of the distributions rather than their means, as illustrated in Figure 4 (left). This manipulation was again expected to affect the likelihood of splitting or merging perceptual categories. Specifically, the High variance condition resulted in greater overlap between the alternative and baseline distributions as compared to the Low variance condition, leading to the prediction that estimates of baseline trials in the High variance condition would be regularized downward more than in the Low variance condition.

### Materials and methods

#### Participants

Fourteen students participated in the experiment for course credit or monetary compensation ($10). All subjects gave informed consent and the study was approved by the Princeton University Institutional Review Board. No subjects were excluded from Experiment 2.

#### Procedure

The procedure was identical to Experiment 1, except that the alternative trials differed in their standard deviations. Both High and Low variance alternative trials had a mean of 35; High variance trials had a standard deviation of 20, while Low variance trials had a standard deviation of 10. Baseline trials (same for both conditions) had a mean of 65 and a standard deviation of 20.

### Results and discussion

The average responses on baseline trials in each condition are shown in Figure 4 (right). Judgments of the number of circles on baseline trials in the High variance condition (mean = 59.75) were significantly lower than in the Low variance condition [mean = 61.82, *t*_(13)_ = 2.72, *p* < 0.05, *d* = 0.60]. This result is consistent with the hypothesis that participants were more likely to merge the alternative and baseline distributions together in the High variance condition than in the Low variance condition. While we observed differential regularization across conditions, these estimates individually were both significantly different from the true average [High variance: *t*_(13)_ = 4.49, *p* < 0.001, *d* = 1.20; Low variance: *t*_(13)_ = 3.30, *p* < 0.01, *d* = 0.88].

We also examined the judgments on alternative trials. In this case, the effect of regularization was symmetric and the average number of circles reported by participants deviated significantly from the true average in the direction of the baseline average: 39.64 for the High variance condition [*t*_(13)_ = 6.07, *p* < 0.001, *d* = 1.62] and 37.45 for the Low variance condition [*t*_(13)_ = 3.39, *p* < 0.01, *d* = 0.91]. Furthermore, the deviation was greater for the High variance condition than for the Low variance condition [*t*_(13)_ = 2.63, *p* < 0.05, *d* = 0.70], consistent with our hypothesis that category merging (and hence more regularization) is more likely to occur in the High variance condition. From a theoretical perspective, the difference between these results and those of Experiment 1 can be explained by the idea that with a larger standard deviation category merging is more likely for both High and Low alternative blocks.

We next performed the sequential correlation analysis described in Experiment 1, calculating the correlation between estimates on each baseline trial and the preceding alternative trial. Recall that if estimates are influenced by recently experienced trials, then the correlation should be positive, but only if both trials are assigned to the same merged category. Fisher z-transformed correlations were significantly greater than 0 in the High variance condition [*t*_(13)_ = 2.30, *p* < 0.05, *d* = 0.62] but not in the Low variance alternative condition (*p* = 0.42). We also examined the influence of baseline trials on subsequent alternative trials: Fisher z-transformed correlations were significantly greater than 0 in the High variance condition [*t*_(13)_ = 2.64, *p* < 0.05, *d* = 0.71] but not in the Low variance condition (*p* = 0.89). Consistent with the results of Experiment 1, these results support the hypothesis that the High variance condition promoted category merging while the Low variance condition did not.

## Experiment 3

In both Experiments 1 and 2, the alternative means were lower than the baseline mean. In Experiment 3, we used the same manipulation as in Experiment 1 to examine whether the same effects would be found when the alternative means were higher than the baseline mean. Here we predicted that participants would be more likely to merge the baseline and alternative categories in the Low mean alternative condition in which the two distributions are more similar, than in the High mean alternative condition; accordingly, baseline estimates should be regularized upward to a greater extent in the Low mean alternative condition.

### Materials and methods

#### Participants

Twenty-three students participated in the experiment for monetary compensation ($10). All subjects gave informed consent and the study was approved by the Princeton University Institutional Review Board. Two subjects were excluded from analyses due to their large estimation errors (greater than two standard deviations from the mean across all three experiments).

#### Procedure

**Figure 5:**
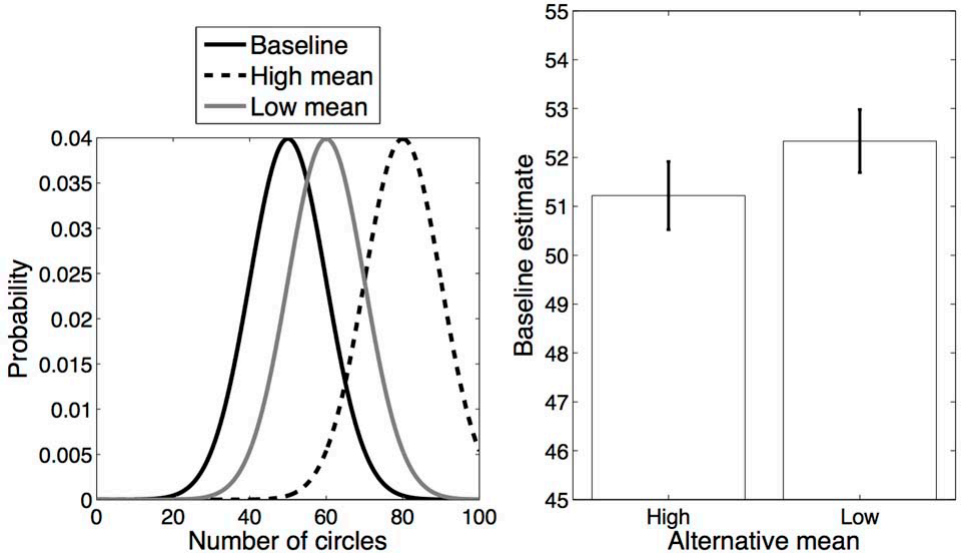
**Experiment 3 design and results**. **Left**: Distributions for each category. **Right**: Average estimates for the baseline category in each condition. Error bars represent standard error of the mean.

The procedure in this experiment was identical to the procedure used in Experiment 1, with only the category means changed. Specifically, we used the following category means: 50 for the baseline trials, 60 for alternative trials in the Low mean alternative condition, and 80 for alternative trials in the High mean alternative condition (see Figure 5, left).

### Results and discussion

The average responses on baseline trials in each condition are shown in Figure 5 (right). Estimates of the number of circles on baseline trials in the High mean alternative condition (mean = 51.08) were significantly lower than in the Low mean alternative condition [mean = 52.37; *t*_(20)_ = 2.67, *p* < 0.05, *d* = 1.19]. The baseline estimates differed significantly from the true average in the Low mean condition [*t*_(20)_ = 3.87, *p* < 0.001, *d* = 1.73], but not in the High mean condition (*p* = 0.13). These results are consistent with the hypothesis that participants were more likely to merge the alternative and baseline distributions together in the Low mean alternative condition (due to greater distributional overlap) than in the High mean alternative condition.

We next examined the estimates on alternative trials. In the High mean condition, the average estimate was 74.23, significantly lower than the true average 80 [*t*_(20)_ = 8.62, *p* < 0.0001, *d* = 3.85]. In the Low mean condition, the average estimate was 58.76, also significantly lower than the true average 60 [*t*_(20)_ = 2.14, *p* < 0.05, *d* = 0.96]. Thus, as in Experiment 2, we found evidence for regularization effects in the alternative estimates, but contrary to our predictions, the effect in the Low mean condition was significantly smaller than the effect in the High mean condition [*t*_(20)_ = 7.28, *p* < 0.0001, *d* = 3.26]. One consideration in interpreting this pattern of results is Weber's law: Discriminability of two numbers decreases with their magnitude, a phenomenon known as the *numerical size effect* (Moyer and Landauer, 1967; Restle, 1970). This might occur, for example, if observers use a logarithmic representation of magnitude. Weberian compression makes it difficult to interpret regularization effects on the alternative trials purely in terms of Occam's razor. In particular, Weberian compression predicts stronger regularization for larger numerical magnitudes, as observed in our experiment. Since the baseline trials are smaller magnitude, they are less affected by Weberian compression, thus licensing our interpretation of the baseline effects in terms of Occam's razor.

Finally, we performed the sequential correlation analysis described in Experiment 1, calculating the correlation between estimates on each baseline trial and the preceding alternative trial. Here Fisher z-transformed correlations were not significantly greater than 0 in the High mean condition (*p* = 0.20) or the Low mean condition (*p* = 0.32). We also examined the influence of baseline trials on subsequent alternative trials. In this case, Fisher z-transformed correlations were significantly greater than 0 in both the High mean condition [*t*_(20)_ = 4.60, *p* < 0.001, *d* = 2.06] and in the Low mean condition [*t*_(20)_ = 4.36, *p* < 0.001, *d* = 1.95]. In contrast with the results of Experiment 1, these results do not support the hypothesis that regularization effects operate on a timescale shorter than an entire block. It may be the case that there is a trade-off between regularization effects that occur on different timescales; the regularization effects on the alternative trials in the current experiment may interact with the trial-by-trial correlations. The idea is that if subjects show stronger “local” (trial-by-trial) regularization, they will show weaker “global” (block-wise) regularization effects, and vice versa. However, our experiment was not designed to test this hypothesis directly.

## A rational analysis

In this section, we frame our experimental results in terms of a Bayesian computational model of the estimation task. This model constitutes a “rational analysis” (Anderson, 1990)—a specification of how an ideal observer would perform in our task. Although we do not necessarily believe that humans are precisely implementing Bayesian inference,^1^ this analysis allows us to explore rather subtle hypotheses about cognitive processes, as we describe below.

According to the Bayesian framework (described formally in the next section), the computational problem facing a participant is to infer the posterior distribution over the number of circles *x_t_* on trial *t*, given noisy sensory input *y_t_*, the circle color *c_t_*, and the history of past trials. For a complete mathematical specification, we make several assumptions about the data-generating process. In particular, both the circle color and number are assumed to be governed by a latent perceptual category *z_t_* drawn from some unknown number of categories. Thus, according to our rational analysis, the participant must implicitly average over her uncertainty about the latent categories in making her estimates. Importantly, we do not impute to the participant a fixed set of categories; rather, both the number and properties of the categories are inferred by the participant from her observational data. The simplicity principle enters into this model via the prior over categories: All else being equal, the model has a preference for a small number of categories.

### Generative process

The starting point of our rational analysis is the specification of a joint distribution over all the variables (both latent and observed) involved in the experimental task. This joint distribution is sometimes known as a *generative model*, since it represents the participant's (putative) assumptions about the process by which the observations were generated. The generative model we assume is a *mixture model*, where the number of circles *x_t_* is drawn from a Gaussian distribution associated with the perceptual category *z_t_* = *k* active on trial *t* (we will use *z_t_* and *k* interchangeably below to indicate categories, with the former used when categories on different trials need to be distinguished). The distribution over *x_t_* is parameterized by a category-specific mean μ_*k*_ and standard deviation σ_*k*_. The observed number of circles *y_t_* (the noisy sensory signal) is drawn from a Gaussian distribution with mean *x_t_* and standard deviation σ_*y*_. Finally, the circle color *c_t_* ∈ {1,…, *C*} is drawn from a category-specific multinomial distribution specified by parameters θ_*k*_. In our experiments, *C* = 3.

We assume that participants begin each block with a prior belief about the parameters of the task μ_*k*_, σ_*k*_ and θ_*k*_ (we assume that the sensory noise σ_*y*_ is fixed). Specifically, we assume a normal-inverse-gamma prior on (μ_*k*_, σ^2^_*k*_): 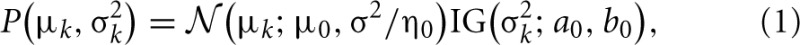 where IG(·; *a*_0_, *b*_0_) is the probability density function of the inverse gamma distribution (see Gelman et al., 2004), and a symmetric Dirichlet distribution prior with parameter λ for the multinomial parameters for the color feature.

To complete the generative model, we need to specify a prior distribution on the set of category assignments, **z**_1:*t*_ = {*z*_1_,…, *z_t_*}, which can be understood as a partition of the observations into latent categories. We want to impute to the participant a prior that is flexible enough to entertain an unbounded number of possible categories, but nevertheless prefers to categorize trials into as few categories as possible. For this purpose, we choose the *Chinese restaurant process* (Aldous, 1985; Pitman, 2002), a prior over an unbounded number of partitions (see Gershman and Blei, 2012, for a tutorial introduction). The name of this prior comes from the following metaphor: Imagine a Chinese restaurant with an unbounded number of tables (categories). The first customer (trial) enters and sits at the first table. Subsequent customers sit at an occupied table with probability proportional to how many people are already sitting there, and at a new table with probability proportional to α ≥ 0 (termed the “concentration” parameter). Once all the customers are seated, one has a partition of trials into categories.^2^ Formally, the Chinese restaurant process prior is given by:

(2) $$\begin{array}{l} P\text{​}\left(z_{t}=k|z_{1:t\;-\;1}\right)=\{\begin{array}{ll} \frac{M_{k}}{t-1+\alpha } & \text{ if }k\text{ is an old category}\\ \frac{\alpha }{t-1+\alpha } & \text{ if }k\text{ is a new category} \end{array} \end{array}$$

where *M_k_* is the number of trials generated by category *k* up to trial *t* (the first trial, at *t* = 1, is by default generated by the first category *k* = 1). The value of α controls the prior belief about the number of categories. As α → 0, all trials will tend to be assigned to the same category; in contrast, as α → ∞, each trial will be assigned to a unique category (the latter limiting case is closely related to exemplar models, as will be described below). The Chinese restaurant process prior was independently discovered by Anderson (1991) in the development of his rational model of categorization, and since then has been used in a wide variety of psychological models (e.g., Gershman et al., 2010; Kemp et al., 2010; Sanborn et al., 2010).

### Posterior inference

Two computational problems face the observer. The first is to infer the posterior distribution over latent perceptual categories given a set of observations. This is done by inverting the generative model using Bayes' rule. The second is to use this distribution to estimate the “true” number of circles on the current trial (*x_t_*) given noisy sensory input (*y_t_*). Note that in our experiments all uncertainty about *y_t_* disappears after feedback (i.e., when *x_t_* is observed). The posterior computations below reflect probabilistic beliefs after feedback is observed. In the Appendix, we describe how predictions are computed before feedback, which we use to predict participants' behavior.

The posterior over categories is stipulated by Bayes' rule:

(3) $$\begin{array}{l} P\text{​}\left(z_{t}|c_{1:t},x_{1:t}\right)\propto \sum_{z_{1:t-1}}P\text{​}\left(c_{t}|z_{1:t},c_{1:t-1}\right)\text{​}P\text{​}\left(x_{t}|z_{1:t},x_{1:t-1}\right)P\text{​}\left(z_{t}|z_{1:t-1}\right)\text{​}P\text{​}\left(z_{1:t-1}\right)\text{​}. \end{array}$$

Using the shorthand *k* = *z_t_* and *c* = *c_t_*, the conditional distributions are given by:

(4) $$\begin{array}{l} P\text{​}\left(c_{t}|z_{1:t},c_{1:t-1}\right)=\int_{\theta }P\text{​}\left(c_{t}|\theta ,z_{1:t},c_{1:t-1}\right)\text{​}d\theta =\frac{\lambda +N_{ck}}{C\lambda +M_{k}} \end{array}$$

(5) $$\begin{array}{l} P\text{​}\left(x_{t}|z_{1:t},x_{1:t-1}\right)=\int_{\mu }\int_{\sigma^{2}}P\text{​}\left(x_{t}|\mu ,\sigma^{2},z_{1:t},x_{1:t-1}\right)\text{​}d\mu d\sigma^{2}=T_{2a_{k}}\left(\frac{x_{t}-{\hat{\mu }}_{k}}{\beta_{k}}\right) \end{array}$$

where *T*_2__*a*_*k*__(x) denotes the student *t*-distribution with 2_*a*_*k*__ degrees of freedom, and

(6) $${\hat{\mu }}_{k}=\frac{\eta_{0}\mu_{0}+M_{k}{\bar{x}}_{k}}{\eta_{k}},\;$$

(7) $$\eta_{k}=M_{k}+\eta_{0},$$

(8) $$a_{k}=M_{k}+\frac{a_{0}}{2},$$

(9) $$b_{k}=b_{0}+\frac{1}{2}\sum_{i=1}^{t-1}\delta \left[z_{i},k\right]{\left(x_{i}-{\bar{x}}_{k}\right)}^{2}+\frac{M_{k}\eta_{0}\text{​}{\left(\mu_{0}-{\bar{x}}_{k}\right)}^{2}}{2\eta_{k}},$$

(10) $$\beta_{k}=\frac{b_{k}\text{​}\left(1+\eta_{k}\right)}{a_{k}\eta_{k}}.$$

Here δ[·,·] = 1 if its arguments are equal, and 0 otherwise. *N_ck_* is the number of times category *k* was presented in conjunction with color *c* and ${\bar{x}}_{k}$ is the average number of circles observed for category *k*. These equations were derived from standard properties of the conjugate-exponential family of probability distributions (Gelman et al., 2004).

Intuitively, Equation 4 keeps track of counts: The posterior *P*(*c_t_*|**z**_1:*t*_, **c**_1:*t* − 1_) will tend to concentrate around the color that was observed most often in conjunction with *z_t_* (conditional on a particular instantiation of **z**_1:*t*_). The parameter λ regularizes the posterior toward the uniform distribution, taking into account the observer's prior uncertainty about the relationship between categories and colors. Similarly, Equation 5 keeps track of category averages: The posterior *P*(*x_t_*|*z_t_*, **z**_1:*t* − 1_, **x**_1:*t* − 1_) will tend to concentrate around the average number of circles observed in conjunction with *z_t_*.

The Bayes-optimal estimator of the number of circles *x_t_* given noisy sensory input *y_t_* is the posterior mean:

(11) $$E\left[x_{t}|y_{t},x_{1:t-1},c_{1:t}\right]\;=\sum_{z_{1:t}}\int_{x}xP\text{​}\left(x_{t}=x,z_{1:t}|y_{t},x_{1:t-1},c_{1:t}\right)\text{​d}x.$$

The estimated number of circles follows a mixture of Gaussians, where the mean of each mixture component is a weighted combination of the category mean and the sensory input.

Because the sum in Equation 3 is intractable to compute exactly, we resort to approximation methods. In the Appendix, we describe a particle filter algorithm (Freitas and Gordon, 2001) for approximating the posterior with a set of samples. While this algorithm can be understood as a provisional hypothesis about how humans might approximate Bayes' rule in this task, it should be emphasized that our data do not directly discriminate between this hypothesis and other types of approximations.

### Model-fitting and comparison to data

We cannot know what sensory input (*y_t_*) a participant is receiving on each trial, so we made the expedient choice (following Huttenlocher et al., 1991, 2000) of setting *y_t_* = *x_t_*, which should be true on average, assuming participants are not systematically biased. We reenter the data by subtracting the empirical mean (true number of circles on average) from all the perceptual estimates, and therefore use μ_0_ = 0. We set λ = 1 and *b*_0_ = 10 (which sets the scale of σ^2^_*k*_), fitting the remaining parameters (α, a_0_, η_0_, σ_*y*_) using a hill-climbing algorithm. Each participant's data were fit with an independent set of parameters. Our objective function was the mean-squared error between the particle filter predictions (Equation A2 in the appendix) and participants' estimates. This is equivalent to the assumption that behavioral responses are normally-distributed around the model predictions; the parameter values minimizing the objective function are thus maximum likelihood estimates.

**Figure 6:**
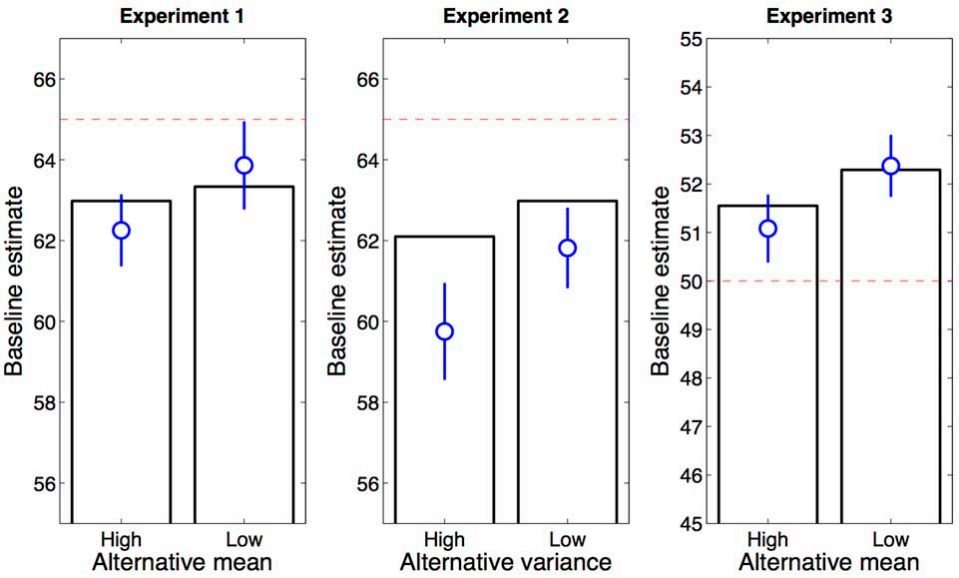
**Model predictions**. Estimates are derived from the fitted rational model for Experiment 1 (**Left**), Experiment 2 (**Middle**), and Experiment 3 (**Right**). Blue circles show the average guess across trials and participants (error bars represent standard error of the mean). Dashed red lines indicate the true average number of circles.

Figure 6 shows the fitted model predictions for the baseline color in Experiments 1–3 (bars) as compared to participants' empirically measured guesses (circles). While not in perfect quantitative agreement with the behavioral data, the model reproduces the observed qualitative pattern. These effects arise in the rational model due to the fact that greater overlap between the baseline and alternative distributions increases the probability that trials with different-colored circles will be attributed to the same category, thereby pushing estimates toward the aggregate mean of the two distributions.

Note that the rational model does not (by construction) account for trial-by-trial correlations, since it assumes that trials are exchangeable, that is, that their order is inconsequential. One could, in principle, extend the model to capture dependencies between trials, but we aimed to keep the model as simple as possible so long as it captured the main results pertaining to the overall accuracy of guesses on baseline trials.

### Comparison to alternative models

The rational model we presented can be contrasted with a continuum of models that have been considered for perceptual estimation tasks. At one pole of the continuum is the model of Huttenlocher et al. (1991), which, in the context of our experiments, endows each color with its own category prototype. The perceptual estimate on a given trial is assumed to be regularized toward the mean associated with the color on that trial (see also Huttenlocher et al., 2000; Hemmer and Steyvers, 2009). This model cannot explain our findings, since it predicts that regularization will always be in the direction of the color-specific mean, disallowing perceptual categories that collapse across color (see Sailor and Antoine, 2005). In other words, the model of Huttenlocher et al. (1991) does not accommodate the possibility of adaptive category merging.

At the other pole is the family of exemplar models, which have proven successful in accounting for human categorization, identification and recognition memory (Medin and Schaffer, 1978; Nosofsky, 1986, 1988; Kruschke, 1992). The essential idea underlying these models is that estimates are formed by comparing the current stimulus to a stored set of memory traces (exemplars). Anderson's rational model of categorization (Anderson, 1991) strikes a middle ground between prototype and exemplar models by assigning observations to a small number of clusters.

As was recognized by Nosofsky (1991) in his discussion of Anderson's rational model of categorization, the rational model becomes equivalent to the exemplar model in the limit α → ∞. In this limit, the number of clusters inferred by the model is equal to the number of observations; hence, each cluster corresponds to an episodic memory trace, and Bayesian estimates correspond to averages of these traces in the same fashion as the exemplar model.^3^ In a sense, the exemplar model postulates the least parsimonious representation of the subject's perceptual inputs, since commonalities between observations are not explicitly abstracted.

**Figure 7:**
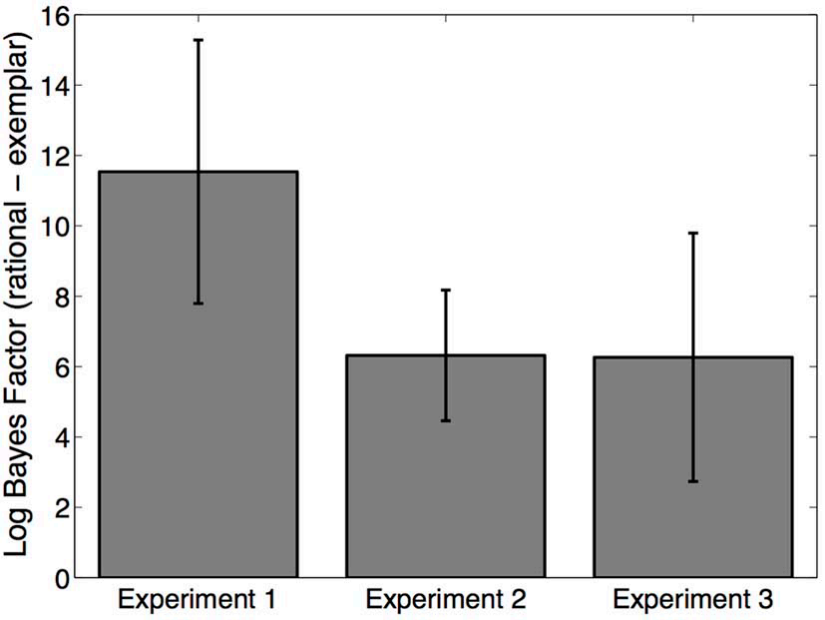
**Model fits**. Log Bayes factor for the rational model relative to the exemplar model. Positive values favor the rational model. Error bars represent standard error of the mean.

It is difficult to rule out an exemplar explanation of our findings through examination of means in each condition. Instead, we undertook a quantitative model comparison to compare our model to the exemplar extreme. First, we compared the evidence for each model on a subject-by-subject basis. Model evidence was quantified by the Bayesian information criterion approximation to the Bayes Factor (Kass and Raftery, 1995), which balances fit to data against model complexity. Note that the rational model has one more parameter (α) than the exemplar model, and is therefore more complex. Model comparison strongly supported the rational model over the exemplar model (Figure 7). A Wilcoxon signed rank test confirmed that the log Bayes factor favored the rational model across all three experiments (*p* < 0.001).

We then used the model fits to investigate the underlying representations posited by the two models. The exemplar model predicts that there should be 20 latent categories (i.e., each observation corresponds to a single latent category). In contrast, the fitted model preferred fewer categories (median = 6), demonstrating that the empirical data are indeed better explained by assuming the simplicity principle.

## General discussion

The experiments reported in this paper bring together two lines of research in cognitive psychology: the “simplicity principle” (a.k.a. “Occam's razor”; Chater and Vitányi, 2003) and the influence of categories on perception (Goldstone, 1995). We show a manifestation in simple perceptual estimates of a simplicity bias toward merging perceptual categories when their statistics are similar: Participants tended to regularize estimates of trials of one color toward those of trials of another color if the stimulus distributions for the two colors had similar means (Experiments 1 and 3) or overlapping tails (Experiment 2).

These findings are consistent with computational models that flexibly infer the number of categories from sensory inputs (Anderson, 1991; Love et al., 2004; Gershman et al., 2010; Sanborn et al., 2010). These models predict that new categories will only be postulated when stimulus statistics differ significantly; otherwise, the stimuli will be merged into a single category. This merging leads to regularization of perceptual estimates, such that perception of a new stimulus will be biased toward the mean of the merged distributions. We presented a rational adaptive categorization model that predicted the qualitative pattern of results and outperformed an exemplar model in terms of explaining the behavioral data. One advantage of using a Bayesian model over simpler models is that it provides a direct link between behavioral phenomena and statistical properties of the environment. As our experiments demonstrated, manipulating these properties leads to systematic changes in behavior that accord with the predictions of the Bayesian model. While mechanistic models (like the exemplar model) can to some extent also fit our data, they do not provide a framework for connecting the effects to environmental properties. This is important, because Bayesian models give us a framework for asking and answering questions at the computational level: What computational problem are humans solving in this task? What statistical assumptions are they making about the problem? How are prior knowledge and sensory evidence being combined? Nonetheless, we have not yet fully mapped out the boundary conditions of the simplicity bias in our task, and so these data should be understood as initial explorations of our model's predictions rather than general statements about Occam's razor in perceptual estimation.

Our results are consistent with other evidence that perception is influenced by unsupervised category learning. Gureckis and Goldstone (2008) asked participants to discriminate between pairs of stimuli that varied along two dimensions, and then in a second phase, trained participants to classify these stimuli into two categories with the classification boundary determined by a single (attended) dimension. The stimuli were designed so that within each category, stimuli fell into two sub-clusters on the basis of the second (unattended) dimension. Despite these sub-clusters being irrelevant for classification, participants were better able to discriminate between stimuli in the same category when they belonged to different sub-clusters. Thus, the underlying cluster structure of the stimuli systematically biased perception.

Although our study used numerical estimation as a paradigm for investigating perceptual biases, we were not interested in estimation *per se*: Only the relative estimation bias between conditions was relevant to our hypothesis. The speeded response requirement made it essentially impossible for participants to explicitly count the number of circles on the screen, thus making past history (in particular, feedback from previous trials) a more influential factor in determining responses compared to the veridical number of circles. Moreover, our study lacked the typical controls used in numerosity experiments (e.g., circle density, area of the region occupied by the circles). Nonetheless, our study may have implications for the study of number perception (Feigenson et al., 2004). In particular, our results suggest that numerical estimation is sensitive not only to the veridical numerosity, but can also be influenced by the distribution of numbers in recent experience. This points toward the existence of a more sophisticated number perception system that incorporates top–down knowledge about numerosity statistics.

We have interpreted our results in terms of Occam's razor, but alternative interpretations may also be possible. For example, an exemplar model (e.g., Nosofsky, 1986; Kruschke, 1992) that interpolates based on similarity between stimuli could also account for our results; however, we showed both quantitatively and qualitatively that the rational model is a better explanation for the empirical data. Another viable alternative is a model in which the stimulus is assumed to have been drawn from one of two distributions (e.g., a mixture of Gaussians). In other words, the participant always assumes two distributions, but has uncertainty about which one generated the data. A potential problem with this account is that it assumes that participants already know the two distributions, whereas we are proposing that they infer them.

A number of questions remain. For example, what are the sequential dynamics of category formation over the course of the experiment? Several previous studies have suggested that sequencing of exemplars plays an important role in unsupervised learning (Anderson, 1991; Clapper and Bower, 1994; Zeithamova and Maddox, 2009), and this factor may also come into play in our task. Although our experiments were not designed to examine this factor directly, we reported significant sequential correlations in Experiments 1 and 2, suggesting that the regularization effects we observed may operate over short timescales. Another question is whether the simplicity bias is itself subject to modulation by task factors. One possibility is that being repeatedly exposed to highly complex environments will lead to a greater tolerance for more complex category structures.

Finally, an important lingering question pertains to the algorithmic implementation of our model. We derived a particle filter algorithm for computing model predictions, and this algorithm has a number of psychologically appealing properties: It is online (processes one data point at a time), it is stochastic (and hence can capture response variability), and it is resource limited (allowing it to emulate cognitive resource limitations). These properties have been discussed at length elsewhere (Brown and Steyvers, 2009; Frank et al., 2010; Gershman et al., 2010; Sanborn et al., 2010). Our experiments were not designed to directly assess these properties or compare the particle filter to other kinds of algorithms, a task we leave to future work. For example, one could ask subjects to perform a secondary task, and examine whether reducing the number of particles can capture the resulting degradation of performance. It is also possible that subjects employ a heuristic algorithm that looks nothing like a particle filter or other formal approximation to Bayesian reasoning (Gigerenzer and Goldstein, 1996). However, we are not aware of heuristic algorithms that could actually perform the task that we gave subjects.

Regardless of the algorithmic implementation, our results demonstrate the importance of Occam's razor in human perceptual estimation. This falls naturally out of a Bayesian analysis of the estimation problem, but such an analysis is really only a starting point for future investigations of the algorithmic and neural computations underlying perception.

### Conflict of interest statement

The authors declare that the research was conducted in the absence of any commercial or financial relationships that could be construed as a potential conflict of interest.

## Footnotes

^1^Nor do we necessarily believe that there is a unique ideal observer, since different priors lead to different inferences, all of which are rational from a statistical point of view.

^2^Note that although the Chinese restaurant process is described in terms of a sequential process, the partitions generated by it are in fact *exchangeable*, meaning that the distribution over partitions is unchanged by permutations of the trial order (Aldous, 1985).

^3^Technically, for this to be true, α must equal 0 when computing predictions with Equation 11

## Appendix

### Particle filtering algorithm

The particle filter is an algorithm that approximates optimal Bayesian inference by updating an approximation to the posterior distribution over the assignment of trials to categories as each observation arrives. This sequential online nature makes it suitable for modeling the dynamics of human learning in our experiments. Similar process models have previously been applied to animal (Daw and Courville, 2008; Gershman et al., 2010) and human (Brown and Steyvers, 2009; Frank et al., 2010; Sanborn et al., 2010) learning, although the generative assumptions of those models differ from our own.^4^

The particle filtering algorithm maintains a set of *L* samples *z*^(1:*L*)^_*t* − 1_ distributed approximately according to the posterior, *P*(*z*_*t* − 1_|**c**_1:*t* − 1_, **x**_1:*t* − 1_). These samples are updated after observing *x_t_* and *c_t_* by drawing *z*^(l)^_t_ for *l* = 1,…, *L* from $P(z_{t}^{\left(l\right)}=k)=\frac{w_{k}}{\sum_{k}w_{k}}$, where

(A1) $$\begin{array}{l} w_{k}=\sum_{l=1}^{L}P\text{​}\left(c_{t}|z_{t}=k,z_{1:t-1}^{\left(l\right)},c_{1:t-1}\right)\text{​}P\text{​}\left(x_{t}|z_{t}=k,z_{1:t-1}^{\left(l\right)},x_{1:t-1}\right)\times P\text{​}\left(z_{t}=k|z_{1:t-1}^{\left(l\right)}\right)\text{​}. \end{array}$$

Drawing samples in this way produces a Monte Carlo approximation to the posterior (Freitas and Gordon, 2001). As *L* → ∞, this approximation will converge to the true posterior.

The particle filter can also be used to estimate the number of circles *x_t_* given noisy sensory input *y_t_* (before feedback):

(A2) $$\begin{array}{l} E\left[x_{t}|y_{t},x_{1:t-1},c_{1:t}\right]=\sum_{z_{1:t}}\int_{x}xP\text{​}\left(x_{t}=x,z_{1:t}|y_{t},x_{1:t-1},c_{1:t}\right)\text{​d}x\\ \;\approx \frac{1}{L}\sum_{l=1}^{L}\frac{\sum_{k}q_{k}^{\left(l\right)}m_{k}^{\left(l\right)}}{\sum_{k}q_{k}^{\left(l\right)}}, \end{array}$$

where

(A3) $$q_{k}^{\left(l\right)}=P\text{​}\left(c_{t}|z_{t}=k,z_{1:t-1}^{\left(l\right)},c_{1:t-1}\right)\text{​}P\text{​}\left(z_{t}=k|z_{1:t-1}^{\left(l\right)}\right)$$

is the posterior weight assigned to category *k* and

(A4) $$\begin{array}{l} m_{k}^{\left(l\right)}=\int_{x}xP\text{​}\left(x_{t}=x|y_{t},z_{t}=k,z_{1:t-1}^{\left(l\right)},x_{1:t-1}\right)\text{​d}x\\ \;=\int_{x}x\frac{P(y_{t}|x_{t}=x)P\text{​}\left(x_{t}=x|z_{t}=k,z_{1:t-1}^{\left(l\right)},x_{1:t-1}\right)}{P\text{​}\left(y_{t},z_{t}=k,z_{1:t-1}^{\left(l\right)},x_{1:t-1}\right)}\text{d}x \end{array}$$

is the prediction of *x_t_* for category *k*. We know of no closed-form expression for *m*^(l)^_*k*_, but we can obtain a very accurate numerical approximation. In our implementation, we set *L* = 100, but the results are not sensitive to this choice.
